# Obstinate Intermittent Coryza—Instantaneous Cure

**Published:** 1852-10

**Authors:** A. Menudier


*Obstinate Intermittent Coryza—Instantaneous Cure.* By Dr. A. Menudier.—A lady, aged twenty-four, had been for some years subject to severe attacks of coryza, coming on twice or three times in a week, and lasting from twelve to thirty-six hours. On one occasion, she was anxious to have the disease arrested immediately. M. Menudier thereupon applied a sinapism from the scapulæ to the loins ; in a quarter of an hour, the coryza began to diminish, and in three quarters of an hour the patient could no longer bear the mustard. The skin was reddened ; but the coryza was cured ; and had not returned at the end of three months.—*London Journ. Med.*

